# Analysis of Mechanical Properties and Fatigue Life of Microturbine Angular Contact Ball Bearings under Eccentric Load Conditions

**DOI:** 10.3390/s23094503

**Published:** 2023-05-05

**Authors:** Haobo Wang, Hangyuan Lv, Zhong Luo

**Affiliations:** 1 Foshan Graduate School of Innovation, Northeastern University, Foshan 528311, China; 2 School of Mechanical Engineering and Automation, Northeastern University, Shenyang 110819, China; 3 Key Laboratory of Vibration and Control of Aero Propulsion Systems Ministry of Education of China, Northeastern University, Shenyang 110819, China

**Keywords:** angular contact ball bearing, theoretical model, finite element method, contact stress, clearance, radial stiffness, fatigue life, preload force

## Abstract

Angular contact ball bearings are common basic components in rotating machinery. During the operation of the bearing, the rolling slips, resulting in contact sliding friction between it and the raceway, which in turn causes wear in the rolling element and increase in the radial clearance of the bearing. The increase in clearance also affects the stiffness of the bearing, which in turn affects the natural frequency and fatigue life of the bearing. At present, there are few studies on the influence of bearing wear (variation of clearance) on life. In this paper, the finite element model based on the theory of contact mechanics is established for the angular contact ball bearing with medium- and high-speed rotation, and the mechanical properties and fatigue life influenced by the internal action of the bearing are analyzed. The effects of radial load and deflection angle on the mechanical properties and fatigue life of the bearing are also studied. Based on the analysis results of bearing contact mechanical properties and clearance changes, the calculation method of bearing life under rolling element wear is established. The influence of the variation of clearance and preload clearance on bearing life is analyzed, and the optimal preload is obtained. The research results of this paper can provide a theoretical basis for optimizing the installation of angular contact ball bearings, reasonably determining the service conditions, and prolonging the service life of bearings, which is necessary for engineering practice.

## 1. Introduction

Angular contact ball bearings are common basic components in rotating machinery. In engineering, due to various unavoidable reasons such as manufacturing and installation errors, variations in load and operating conditions, and performance degradation of parts during service, abnormal wear and even fatigue damage of bearings occurs. The applied load changes the internal force distribution, stress distribution and bearing life of angular contact ball bearings, which is not well analyzed and processed in the existing bearing design and specifications. The research on the mechanical properties and fatigue life analysis methods of angular contact ball bearings under different working conditions has basic support significance for the design, installation and use of bearings, as well as for guiding bearing failure analysis, improving maintenance methods, and improving bearing service life [1,2,3,4].

The static analysis of rolling bearings usually uses the elastic contact deformation between the bearing rolling element and the raceway to establish the inner race balance equation to calculate the displacement of the bearing inner race, and then calculates the load distribution of the bearing rolling element and the change trend of the contact angle through the inner race displacement. However, the static modeling process ignores the influence of bearing components such as bearing cages and sealing races.

Hertz first studied the elastic deformation and stress of the ellipsoidal elastomer and obtained a theoretical solution to the point contact problem with a very small contact size. The Hertz elastic contact theory can calculate the contact problem inside the bearing and has been used until now [5]. Stribeck [6] first applied the Hertz contact theory to rolling bearings and analyzed the relationship between the maximum load and the radial load of the rolling element. Palmegren [7] simplified the Hertz contact calculation, improved the static theory of rolling bearings on this basis, and analyzed the bearing deformation and load under combined load.

Many scholars have carried out in-depth research on the static load distribution and contact problem of rolling bearings. Jones [8] analyzed the load distribution of ball bearings under external loads, and the contact stress and deformation of inner and outer raceways. Zhao [9] and Ricci [10] studied the change of contact angle and contact stress of bearings under axial force. Liao [11] analyzed the variation of the contact stress of the bearing under external load. Tang [12] studied the bearing contact problem via the finite element method. The finite element results were compared with the results obtained with the Hertz theory to verify the feasibility of the finite element method. Xu [13] studied the static contact characteristics of angular contact ball bearings under radial and axial loads, and analyzed the variation law of bearing contact stress. Fang [14] simplified the existing mechanical model by using the internal contact angle of the bearing as an iterative variable and analyzed the influence of various factors on the contact angle under combined load. Liu [15] proposed a numerical iteration method based on the Hertz contact theory to study the load distribution, deformation and stiffness characteristics of ball bearings under combined loads. The emergence of the statics theory provides an important theoretical basis for the design and optimization of bearing structure.

Theoretical and experimental studies have shown that the geometric parameters of the bearing significantly affect the life and other characteristics of the bearing. The finite element model of cylindrical roller bearing heat-stress coupling is established by ANSYS and analyzes the influence of the convex value on bearing temperature rise and contact stress, and the influence of radial load and rotation speed on the optimal convex value [16]. The calculation model of contact stress between roller and raceway and the center trajectory of inner race is established based on the theory of thick-walled cylinders considering the diameter error of the roller and the interference of inner and outer races [17]. The accurate calculation of the force of each roller is realized via numerical calculation. The relationship between the fit size of the bearing inner race and shaft, and the interaction force between them is deduced [18].

During the operation of the bearing, the rolling slips, resulting in contact sliding friction between it and the raceway, which in turn causes wear in the rolling element and increase in the radial clearance of the bearing. The increase in clearance also affects the stiffness of the rotor bearing, which in turn affects the natural frequency and fatigue life of the bearing. Considering the influence of the bending elastic deformation of the outer race, the bending deformation of the bolt shaft, the modification of the needle roller and the radial working clearance on the needle roller load, the mechanical model of the bolt roller needle roller bearing was established [19]. The stress distribution of the needle roller bearing was obtained via numerical calculation, and the relationship between the maximum contact stress of the maximum needle roller and the raceway, the contact fatigue life and the load were established, and the numerical solution of the effective rated static and dynamic load was obtained. On the basis of the L–P life theory, considering the influence of roller life and load distribution in the length direction of roller on the overall life of the bearing, the calculation method of the basic rated life of roller bearing is established [20]. Considering the influence of interference fit and temperature on the circumferential stress inside the race, a modified calculation method of bearing life based on the maximum shear stress of the subsurface layer is established. A three-dimensional finite element model of thin-walled deep-groove ball bearings is established based on ANSYS software [21].

Zhang [22] established a mechanical model for analyzing the ball-raceway contact state of high-speed ball bearings based on the influence of external load, rotational speed and preload on the ball-raceway contact state. The relationship between preload and bearing life is given. Zhang [23] analyzed the influence of radial load, eccentric load angle and interference on the mechanical properties and fatigue life of the bearing. Xu [24] showed the impact of clearance on the vibration characteristic of the bearing. Xiao [25] established the finite element model of double-row tapered roller bearings, calculated the contact load between roller and raceway, and analyzed the influence of axial clearance on contact load. Considering the influence of bending deformation of outer race on ball load distribution after loading, the influence of load, shaft-hole fit clearance and ball number on ball load and bearing life is analyzed. At present, there are few studies on the influence of bearing wear (clearance change) on the life of angular contact ball bearings.

In this paper, the quasi-static method is used to analyze and calculate the contact mechanical state of angular contact rolling bearings under the conditions of high speed and large load. The contact mechanical characteristics of angular contact bearings are calculated from the variation of bearing clearance, and a calculation method of bearing life about rolling elements wear is established, so as to analyze and evaluate the operation state of the bearings and provide a reference for the rational use of angular contact bearings in practical engineering applications.

## 2. Structure and Installation of Angular Contact Ball Bearings

**Figure 1:**
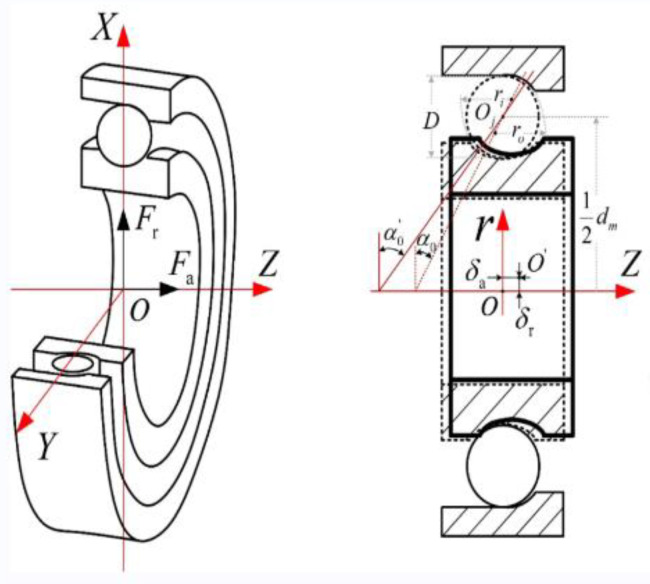
Angular contact ball bearing.

In this paper, one of the angular contact ball bearings in the air turbine is taken as an example for study. The bearing accuracy level is P4, and the structure is shown in Figure 1. The main structural parameters are shown in Table 1.

**Figure 2:**
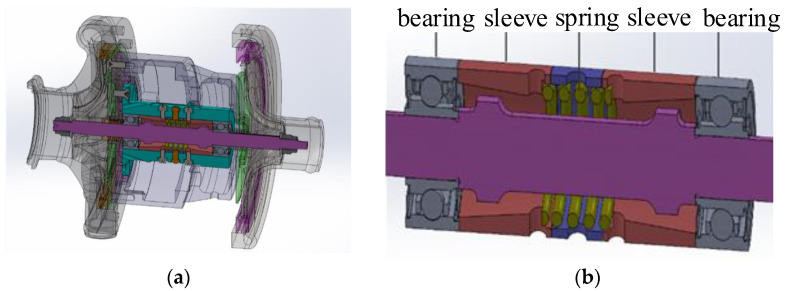
Mounting of angular contact ball bearings on turbine rotors: (**a**) structure design of turbine; (**b**) installation of bearings on turbine rotors.

The materials of races and balls are Cr4Mo4V, which has good material processing performance and high temperature performance. The hardness of the material is 58 HRC at 316 °C for a long time. The cage material is tin bronze QSn6.5-0.1, which has high strength, elasticity, wear resistance, diamagnetism and corrosion resistance. The sealing material is fluorine rubber (PECK). The installation of the bearing on the turbine rotor is shown in Figure 2.

It can be seen that, when installing the turbine end angle contact ball bearing, the combination of positioning preload and constant pressure preload is adopted in Figure 2. Through the test on the rig, the outer surface temperature of the bearing outer race is measured to be about 120° during operation. The load of the turbine end angular contact ball bearing is measured with the high-temperature strain gauge race under several typical working conditions:

1. 57,000 r/min, the radial force of the bearing is 160 N and the axial force is 50 N;
2. 63,000 r/min, bearing radial load is 150 N, axial load is 70–170 N;
3. 68,000 r/min, bearing radial load is 90 N, axial load is 130 N.

The second condition is the most important. The turbine runs for a long time under this condition. The following is the analysis of the optimal axial installation preload of the bearing for the second condition.

## 3. Analysis Method of Internal Load and Life of Angular Contact Ball Bearing

The initial contact angle of the angular contact ball bearing is 15°. According to the force and torque balance equations for the bearing inner race established by Jones [26], as shown in Equations (1)–(3), solving these equations can obtain the radial displacement of the bearing *δ_r_*, axial displacement *δ_a_* and preload angle *θ*.

(1) $$\begin{array}{l} F_{a}=K_{n}A^{\frac{3}{2}}\sum_{\psi =0}^{\pm \pi }\frac{{\left\{{\left[{\left(\sin\alpha^{0}+\bar{\delta_{a}}+R_{i}\bar{\theta }\cos\psi \right)}^{2}+{\left(\cos\alpha^{0}+\bar{\delta_{r}}\cos\psi \right)}^{2}\right]}^{\frac{1}{2}}-1\right\}}^{\frac{3}{2}}}{{\left[{\left(\sin\alpha^{0}+\bar{\delta_{a}}+R_{i}\bar{\theta }\cos\psi \right)}^{2}+{\left(\cos\alpha^{0}+\bar{\delta_{r}}\cos\psi \right)}^{2}\right]}^{\frac{1}{2}}}\\ \;\;\;\;\;\times \left(\sin\alpha^{0}+\bar{\delta_{a}}+R_{i}\bar{\theta }\cos\psi \right) \end{array}$$

(2) $$\begin{array}{l} F_{r}=K_{n}A^{\frac{3}{2}}\sum_{\psi =0}^{\pm \pi }\frac{{\left\{{\left[{\left(\sin\alpha^{0}+\bar{\delta_{a}}+R_{i}\bar{\theta }\cos\psi \right)}^{2}+{\left(\cos\alpha^{0}+\bar{\delta_{r}}\cos\psi \right)}^{2}\right]}^{\frac{1}{2}}-1\right\}}^{\frac{3}{2}}}{{\left[{\left(\sin\alpha^{0}+\bar{\delta_{a}}+R_{i}\bar{\theta }\cos\psi \right)}^{2}+{\left(\cos\alpha^{0}+\bar{\delta_{r}}\cos\psi \right)}^{2}\right]}^{\frac{1}{2}}}\\ \;\;\;\;\times \left(\cos\alpha^{0}+\bar{\delta_{r}}\cos\psi \right)\cos\psi \end{array}$$

(3) $$\begin{array}{l} M=\frac{1}{2}d_{m}K_{n}A^{\frac{3}{2}}\sum_{\psi =0}^{\pm \pi }\frac{{\left\{{\left[{\left(\sin\alpha^{0}+\bar{\delta_{a}}+R_{i}\bar{\theta }\cos\psi \right)}^{2}+{\left(\cos\alpha^{0}+\bar{\delta_{r}}\cos\psi \right)}^{2}\right]}^{\frac{1}{2}}-1\right\}}^{\frac{3}{2}}}{{\left[{\left(\sin\alpha^{0}+\bar{\delta_{a}}+R_{i}\bar{\theta }\cos\psi \right)}^{2}+{\left(\cos\alpha^{0}+\bar{\delta_{r}}\cos\psi \right)}^{2}\right]}^{\frac{1}{2}}}\\ \;\;\;\;\;\times \left(\sin\alpha^{0}+\bar{\delta_{a}}+R_{i}\bar{\theta }\cos\psi \right)\cos\psi \end{array}$$

(4) $$\bar{\delta_{r}}=2.857\delta_{r}$$

(5) $$\bar{\delta_{a}}=2.857\delta_{a}$$

(6) $$\bar{\theta }=2.857\theta$$

where $F_{a}$ is axial load; $F_{r}$ is radial load; *M* is torque load. *A* is the distance between the center of curvature of the inner and outer race grooves, $K_{n}$ is the load-deformation coefficient, $R_{i}$ is the motion radius of the center of curvature of the inner groove, ψ is azimuthal angle; *d_m_* is the diameter of pitch circle; $\delta_{a}$ is the axial displacement of the inner race; $\delta_{r}$ is the radial position of the inner race.

The radial stiffness *K*_r_ formula of the bearing is:

(7) $$K_{r}=\frac{F_{r}}{\delta_{r}}$$

Ball bearing fatigue life can be obtained according to the rolling contact load and L–P fatigue life formula [27].

Rated contact dynamic loads of inner and outer raceways:

(8) $$Q_{ci,e}=98.1{\left(\frac{f_{i,e}}{2f_{i,e}-1}\right)}^{0.41}\frac{{\left(1\mp \gamma \right)}^{1.39}}{{\left(1\pm \gamma \right)}^{\frac{1}{3}}}{\left(\frac{\gamma }{\cos\alpha^{0}}\right)}^{0.3}D_{w}{}^{1.8}Z^{-\frac{1}{3}}$$

where, ± and ∓ represent the inner race contact and the outer race contact, respectively. f is the coefficient of curvature radius of ditch; γ is the geometric parameter; $D_{w}$ is the diameter of the ball, *Z* is the number of balls. For the inner race, ± takes + and∓ takes -; Outer races is opposite. *i* and *e* represent the inner and outer races. Then the ball contact load $Q_{j}$ can be obtained.

Equivalent contact load of inner raceway:

(9) $$Q_{ei}={\left(\frac{1}{Z}\overset{j=Z}{\underset{j=1}{\sum }}Q_{j}^{3}\right)}^{\frac{1}{3}}$$

Equivalent contact load of outer raceway:

(10) $$Q_{ee}={\left(\frac{1}{Z}\overset{j=Z}{\underset{j=1}{\sum }}Q_{j}^{\frac{10}{3}}\right)}^{0.3}$$

The Hertz contact theory is used to analyze the stress and deformation of the rolling bearing in contact with the groove.

The relationship of contact load and contact deformation ($\delta_{ij}$, $\delta_{oj}$) between the *j*th rolling element and inner and outer races is:

(11) $$\begin{array}{c} Q_{ij}=K_{i}\delta_{ij}^{3/2}\\ Q_{oj}=K_{o}\delta_{oj}^{3/2} \end{array}$$

where *K_i_* and *K_o_* are load-deformation coefficients between the ball and the inner and outer race respectively.

The contact deformation of the *j*th rolling body with the inner and outer races is

(12) $$\begin{array}{l} \delta_{ij}={\left[{\left(A_{1j}-X_{1j}\right)}^{2}+{\left(A_{2j}-X_{2j}\right)}^{2}\right]}^{1/2}-(f_{i}-0.5)D\\ \delta_{oj}={\left(X_{1j}{}^{2}+X_{2j}{}^{2}\right)}^{1/2}-(f_{i}-0.5)D=0 \end{array}$$

where *A*_1*j*_ is the axial distance of the curvature center of the inner and outer race grooves at the ball position, *A*_2*j*_ is the radial distance between the curvature center trajectories of the groove, and *X*_1*j*_ and *X*_2*j*_ are the intermediate variables related to the contact angle of the inner and outer races, respectively. The relationship between the contact angle of inner and outer races and radial and axial distances and contact deformation is as follows:

(13) $$\left\{\begin{array}{c} \cos\alpha_{oj}=\frac{X_{2j}}{(f_{o}-0.5)D+\delta_{oj}}\\ \sin\alpha_{oj}=\frac{X_{1j}}{(f_{o}-0.5)D+\delta_{oj}}\\ \cos\alpha_{ij}=\frac{A_{2j}-X_{2j}}{(f_{o}-0.5)D+\delta_{ij}}\\ \cos\alpha_{ij}=\frac{A_{1j}-X_{1j}}{(f_{o}-0.5)D+\delta_{ij}} \end{array}\right.$$

When the ball contacts the raceway, the contact area is a semi-ellipsoid with the following long and short axes:

(14) $$a=n_{a}{\left(\frac{3\eta Q}{2\sum \rho }\right)}^{1/3},\;b=n_{b}{\left(\frac{3\eta Q}{2\sum \rho }\right)}^{1/3}$$

where *Q* is contact load, η is comprehensive elastic modulus, *∑ρ* is the main curvature and function of the contact point, *F*(*ρ*) is the main curvature difference function of the contact point, *n_a_*, *n_b_* and *n_σ_* are the coefficients related to the main curvature difference function *F*(*ρ*) of the contact point, which can be obtained from the table with *F*(*ρ*), and *Q*_max_ is the maximum load to a single roller.

The maximum compressive stress at the contact area is:

(15) $$\sigma_{\max}=\frac{3Q_{\max}}{2\pi ab}$$

Rolling bearing fatigue life uses *L*–*P* subsurface fatigue life [12],

(16) $$L_{10}={\left(\frac{C_{a}}{P}\right)}^{\varepsilon }\frac{10^{6}}{60n}$$

where *L*_10_ is the basic rated life with 90% reliability, Hour; *C_a_* is the basic rated dynamic load of the bearing, N; *P* is the equivalent momentum load of the bearing, N; *n* is bearing speed, r/min; *ε* is the bearing life index and *ε* = 3 for the angular contact ball bearing.

## 4. Finite Element Model of Angular Contact Ball Bearings

**Figure 3:**
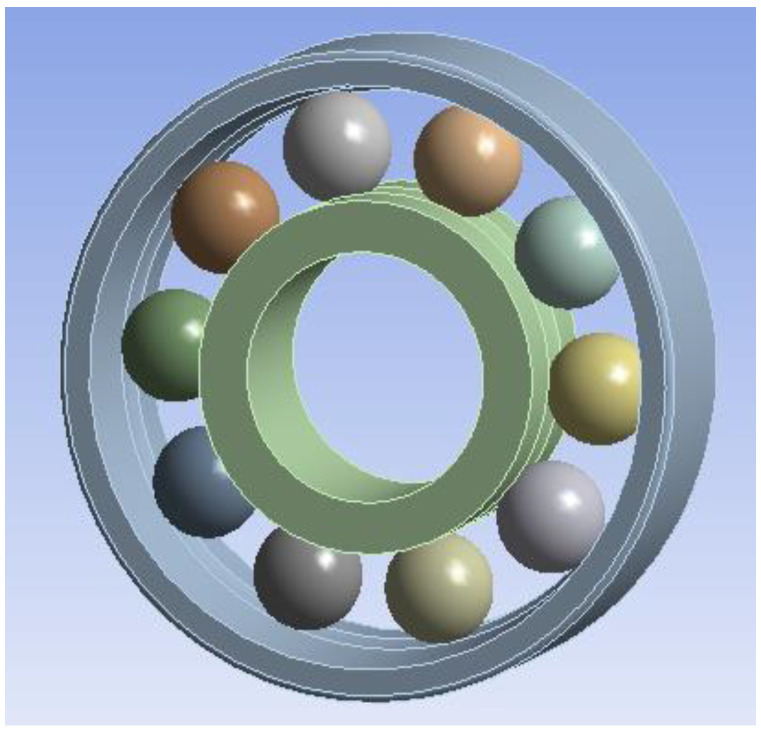
Three-dimensional solid model of bearing.

According to the main structural parameters of the bearing in Table 1, a finite element model is established with SolidWorks. Because the chamfer and edge of the rolling bearing have little effect on the contact stress and deformation of the bearing, they are ignored in modeling. With the quasi-static analysis, the influence of the cage can be ignored [28], and the degree of freedom of the constrained rolling element revolution can be used to simulate the role of the cage. In this paper, ANSYS Workbench is used for finite element analysis. The finite element model is shown in Figure 3. The elastic modulus of the inner race material is 210 GPa and Poisson‘s ratio is 0.3. Impose the following constraints: (1) Fixed constraints are applied on the outer surface of the outer race; (2) The rotation joint is applied on the inner surface of the shaft, and only the translation freedom in the y direction and the rotation freedom in the z direction are retained. (3) The translation freedom of the inner race in the y direction is retained; (4) A cylindrical coordinate system is established to limit the degree of freedom of the rotation of the ball in the direction of revolution.

**Figure 4:**
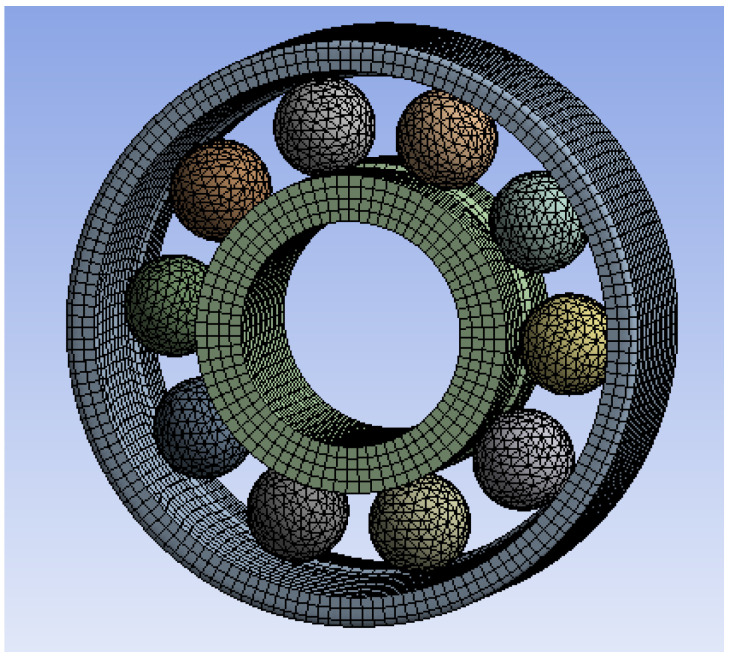
Overall meshing.

The rolling element is divided by tetrahedral mesh, and the inner and outer races are divided by hexahedral mesh. The surface of the inner and outer races and the shaft is divided by mapping surface to make the mesh more uniform and obtain more accurate results. The finite element model meshing generates a total of 105,225 elements and 370,509 nodes. The mesh quality is 0.9, and the overall meshing is shown in Figure 4.

**Figure 5:**
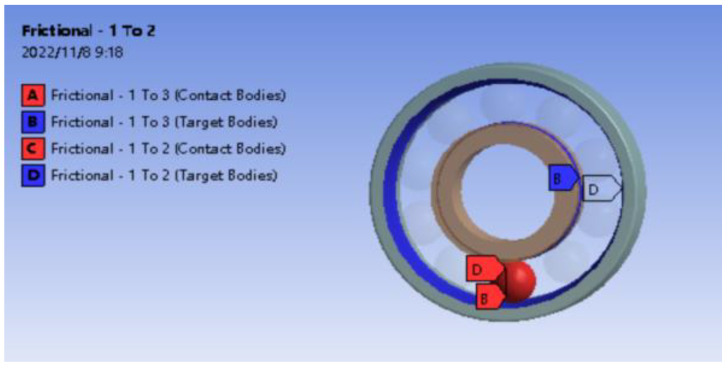
Contact settings of the finite element model.

Contact pairs need to be established when surface-to-surface contact occurs in multi-body parts. The bearing has 10 rolling elements. Each rolling element needs to set contact pairs with inner and outer races. A total of 20 pairs of contacts have been established. The contact between the rolling element and the inner and outer races is friction contact. According to the contact setting, when the concave surface contacts with the convex surface, the convex surface is specified as the contact surface, and the concave surface is the target surface, so the inner and outer raceway surfaces are set as the target surface, and the rolling element surface is the contact surface. The contact friction coefficient is set to 0.15 [23]. The contact between a rolling element and the raceway is shown in Figure 5.

**Figure 6:**
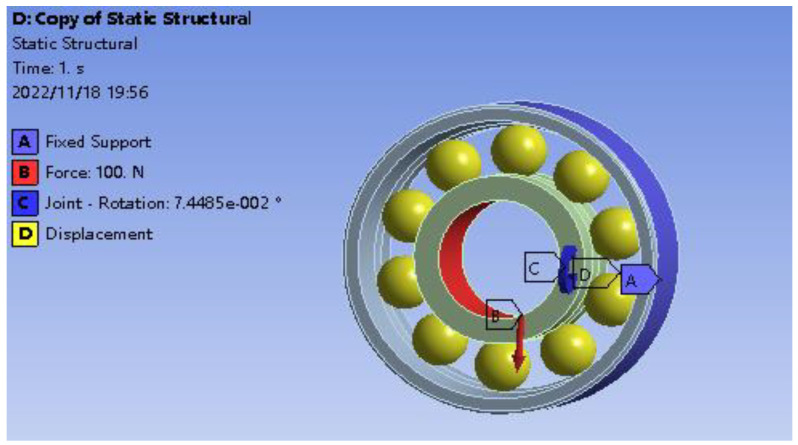
Constraints and load settings of the finite element model.

Considering the working and assembly installation of the bearing, a fixed constraint is added to the outer surface of the outer race to constrain all degrees of freedom of the outer surface of the outer race; a rotating joint is added to the inner surface of the inner race to limit the displacement freedom in the Y and Z directions and only the displacement freedom in the X-axis direction is retained in the cylindrical coordinate system. The rotation degree of freedom is around the X-axis, the angular displacement load is applied to the rotating pair and the direction rotates around the X-axis. It is used to simulate the eccentric load of the inner race without limiting the movement of the inner race in the X-axis. The bearing load is added to the inner surface of the bearing inner race to simulate the radial load of the bearing inner race. In order to facilitate the analysis, the cage is ignored in the overall modeling of the bearing, but it is still necessary to consider the constraint effect of the cage on the rolling. The cylindrical coordinate system is established and the rotation degree of freedom of each rolling element along the revolution direction is limited in the cylindrical coordinate system. The details are shown in Figure 6.

Since the radial stiffness and fatigue life of the mechanical model and the finite element model are obtained by substituting the calculated values of the ball contact load and the radial displacement of the bearing into Equations (7)–(12), the ball contact load and the radial displacement of the bearing are used to verify the finite element model.

**Figure 7:**
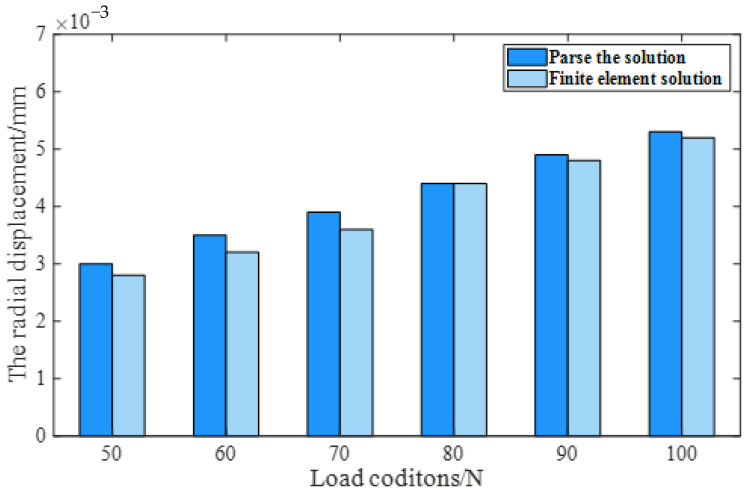
The radial displacement of the angular contact ball bearing under six conditions.

**Figure 8:**
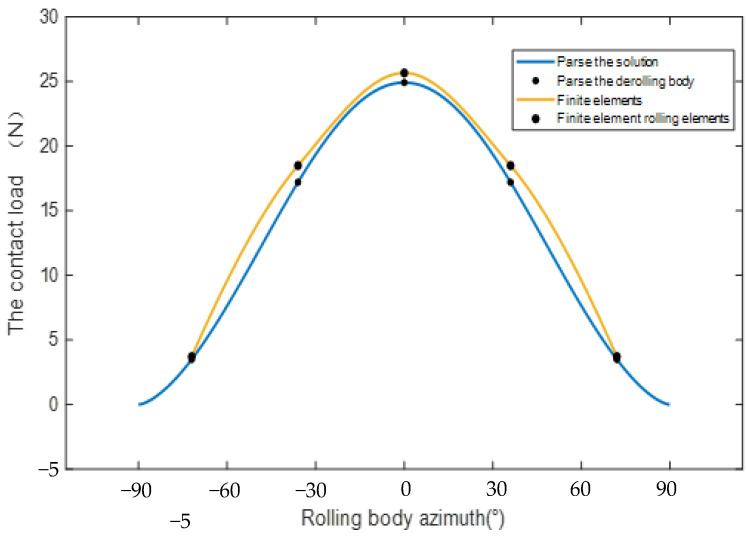
The contact load distribution of the angular contact ball bearing in condition 6.

Based on the finite element model, six conditions are set up, as shown in Table 2. The radial displacement of the bearing under these six conditions is shown in Figure 7. Condition 6 is taken as an example, as shown in Figure 8. In the direction of the radial load, the azimuth angle of the ball is 0° and the azimuth angle ± 90° range is the main bearing ball. The radial displacement is analyzed. The maximum error is 9%, which is within the allowable range of error, indicating the correctness of the finite element model.

## 5. Mechanical Properties and Fatigue Life of Angular Contact Ball Bearings under a Variation of Parameters

### 5.1. Radial Load

**Figure 9:**
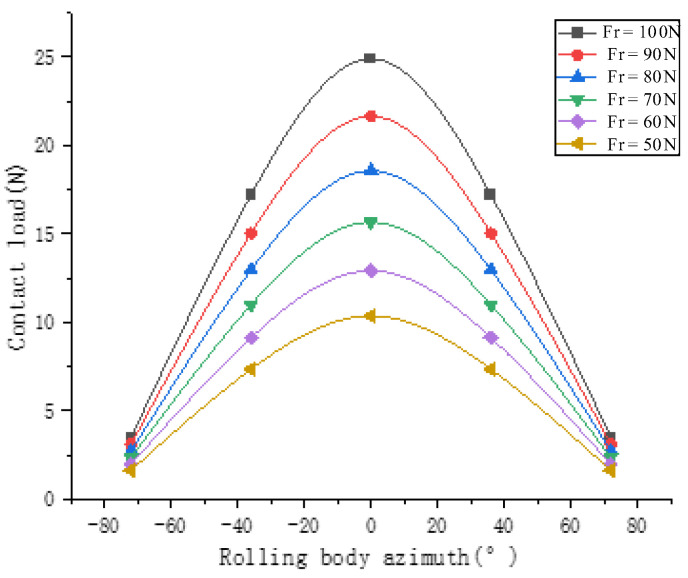
Contact load of angular contact ball bearing.

**Figure 10:**
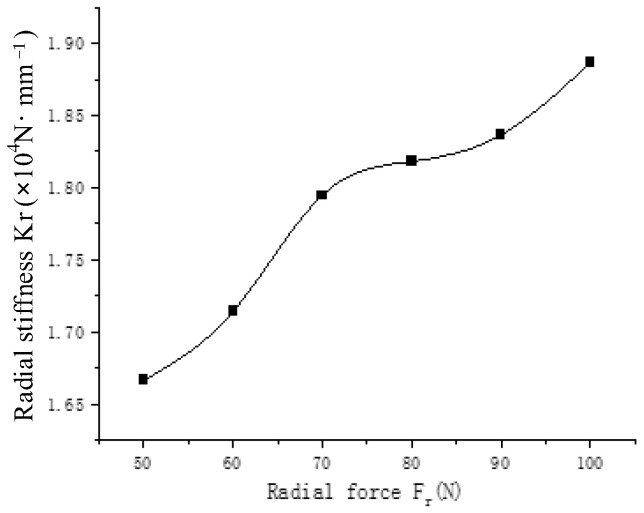
Radial stiffness of angular contact ball bearing.

**Figure 11:**
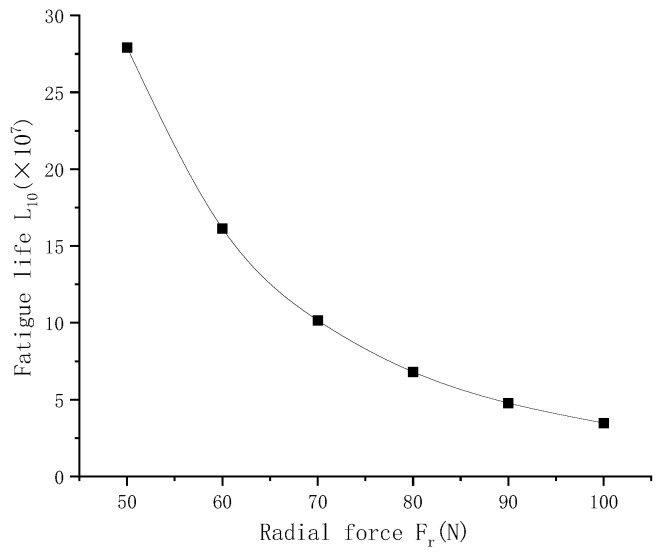
Life of angular contact ball bearing.

**Figure 12:**
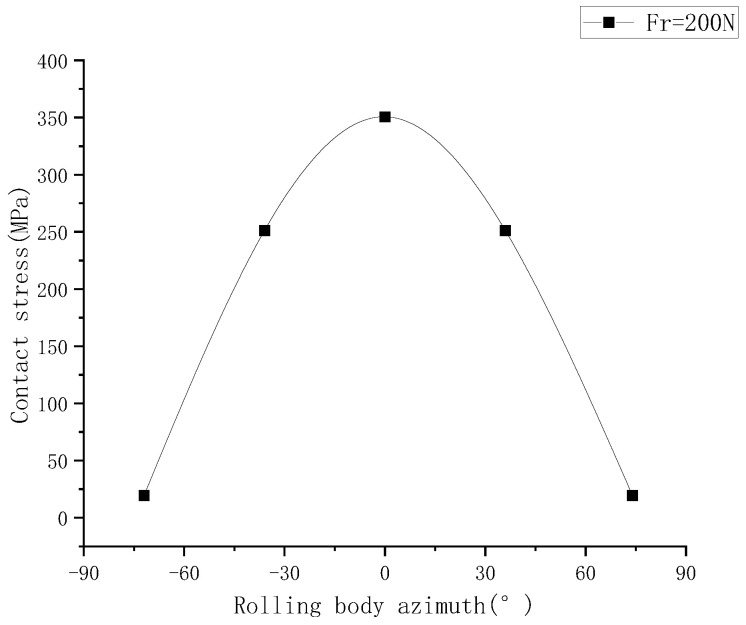
Contact stress between roller and inner race of bearing under condition 6.

Figure 9 shows, from bottom to top, the contact load diagram of the angular contact ball bearing, which is subjected to a radial force of 50 N, 60 N, 70 N, 80 N, 90 N and 100 N. It can be concluded that the contact load of the angular contact ball bearing increases with the increase in radial load. The radial stiffness of the angular contact ball bearing increases with the increase in radial force in Figure 10. The life of the angular contact ball bearing is shown in Figure 11. The fatigue life of the angular contact ball bearing decreases with the increase in radial force, and the decreasing speed is slower and slower. The contact load between the roller and the inner raceway of the angular contact ball bearing under condition 6 is shown in Figure 12.

### 5.2. Deflection Angle

**Figure 13:**
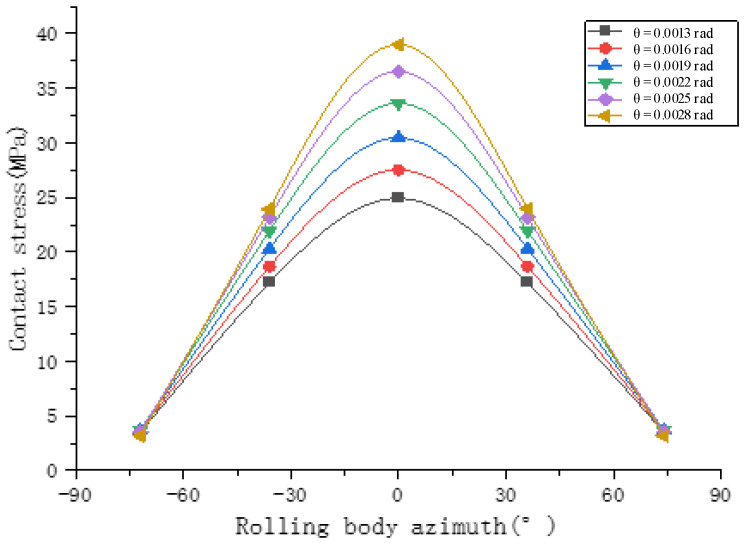
The rolling element load under different deflection angles with 200N of radial load.

**Figure 14:**
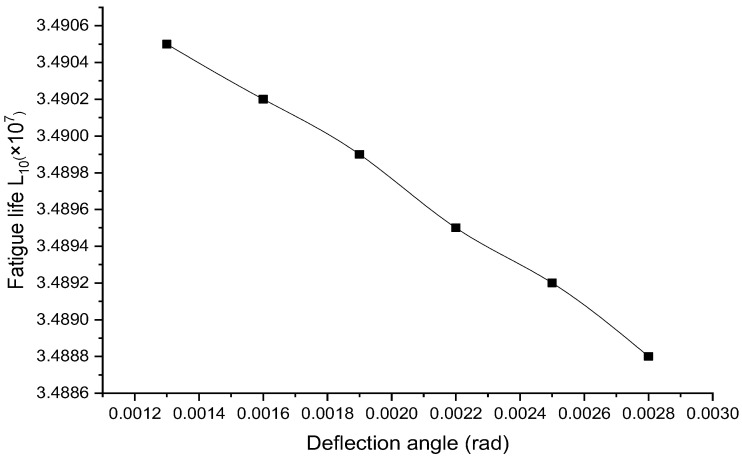
Variation of angular contact ball bearing life with offset angle.

Hybrid loading conditions are considered to analyze the mechanical properties and fatigue life of bearings. The contact load between the rollers and the inner raceway at different deflection angles (0.0013 rad, 0.0016 rad, 0.0019 rad, 0.0022 rad, 0.0025 rad and 0.0028 rad, from top to bottom) under a radial load of 200 N is shown in Figure 13. The stress change slope increases with the increase in the deflection angle. The variation of the angular contact ball bearing life with the preload angle is shown in Figure 14. As the deflection angle increases, the bearing life decreases.

### 5.3. Clearance

According to the theory of elastic wall thickness ring [18], the radial clearance variation of the ball bearing and the initial contact angle of the rolling bearing can be obtained.

Initial contact angle of rolling bearing:

(17) $$\alpha^{0}=\cos^{-1}(1-\frac{u_{r}+\Delta u_{r}}{2A})$$

where *A* is the curvature center distance between the inner and outer race grooves.

(18) $$A=(f_{i}+f_{e}-1)D_{w}=(0.52+0.5235-1)\times 4.85=0.2110\;\mathrm{mm}$$

Here, assuming interference Δ*u_r_* = 0, the initial contact angle of the bearing is:

(19) $$\alpha^{0}=\cos^{-1}(1-\frac{u_{r}+\Delta u_{r}}{2A})=\cos^{-1}(1-\frac{0.0124+0}{2\times 0.2110})=13.9{}^{\circ}$$

When the selected type of angular contact ball bearing is subjected to quasi-static calculation, the speed is considered to be stable and the value is 63,000 r/min. The load of the bearing is as follows: (1) The radial load Fr of the bearing takes the typical value of 150N. (2) The axial load of the bearing depends on the sum of the working load of the turbine disk and the axial preload of the bearing. Through the aerodynamic analysis of the turbine, although the axial load of the turbine is variable, it is generally about 40 N and is unidirectional.

#### 5.3.1. Influence of Clearance on Bearing Stress and Life

The contact state and fatigue life calculation results under a different clearance variation and axial force are shown in Table 3. The clearance is 13.3 mm when the bearing deflection angle is 15 degrees. Hybrid loading conditions are considered here. Under the condition of a radial load of 150 N and an axial load of 40 N, the speed is 1500 r/min. As the clearance increases, the life increases.

#### 5.3.2. The Variation of Stress and Life of Rolling Bearing under Different Preload Force-Clearance

**Figure 15:**
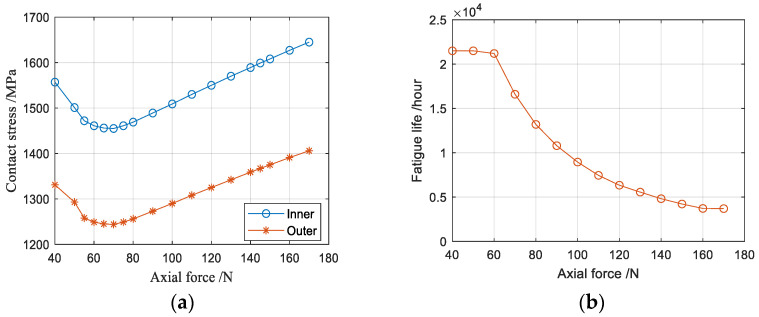
The maximum contact stress and life change of the angular contact ball bearing under different axial forces: (**a**) Stress diagram under different axial forces; (**b**) Life diagram under different axial forces.

Under different preloads, the clearance changes accordingly, which changes the contact angle, stress and bearing life. Setting the radial load at 150 N, the calculation results of the clearance change, contact state and fatigue life of the bearing under different axial force conditions are shown in Table 4. The curves of the maximum contact stress between the rolling elements and the inner race with different axial forces listed in Table 4 are shown in Figure 15a. When the axial force is 70 N, the lowest contact stress value appears. At this time, the corresponding bearing installation preload is 30 N. As shown in Figure 15b, the life value calculated via quasi-statics has no extremum, and the change of fatigue life cannot be used to judge the optimal preload.

## 6. Conclusions

In this paper, the contact mechanical state of the angular contact ball bearing is analyzed and calculated via the quasi-static method under high speed and large load in engineering. The mechanical analysis model and finite element model of the angular contact ball bearing are established under different clearances, and the correctness of the finite element model is verified. The effects of radial load and eccentric load angle on the mechanical properties and fatigue life of the bearing are analyzed. By calculating the mechanical properties of bearing contact and the change of bearing clearance, a bearing life calculation method considering rolling element wear (clearance change) is established. The influence of bearing clearance and preload clearance change on bearing life is studied. The main conclusions are as follows:

- The contact load of the angular contact ball bearing increases with the increase in radial load. When the radial load is 100 N, the maximum contact load is 25 N. With the increase in the deflection angle, the contact stress also increases. The larger the deflection angle, the faster the contact stress increases;
- The radial stiffness of the angular contact ball bearing increases with the increase in radial force; the maximum radial stiffness is reached at 1.9 × 10^4^ N/mm. The life of the angular contact ball bearing decreases with the increase in radial force. When the radial force increases, the bearing life decreases slowly. The bearing life decreases with the increase in the deflection angle, but the overall life change amplitude is small;
- When the radial force is constant, the life increases with the increase in the clearance; the fatigue life increases from 14,254 h to 34,323 h when the radial clearance increases from 12.4 μm to 20.9 μm.
- Considering the clearance, when the axial force is 70 N, the lowest contact stress occurs. At this time, the corresponding bearing installation preload is 30 N.

The research results of this paper can provide a theoretical basis for optimizing the installation of angular contact ball bearings, reasonably determining the use conditions and prolonging the service life of bearings, which is necessary for engineering practice.

## Figures and Tables

**Table 1:** Structural parameters of angular contact ball bearings.

Parameters	Values
Inner race diameter (mm)	10
Outer race diameter (mm)	26
Inner race width (mm)	8
Outer race width (mm)	11.50
Ball diameter (mm)	4.4848
Pitch diameter (mm)	18
Contact angle (°)	15
Coefficient of curvature of outer race (mm)	0.5235
Coefficient of curvature of inner race (mm)	0.52
Outer race shoulder diameter (mm)	21.4
Inner race shoulder diameter (mm)	15.74
Number of balls	10

**Table 2:** Angular contact ball bearing load conditions.

Conditions	Fr/N	Deflection Angle/Rad
1	50	0.007
2	60	0.008
3	70	0.009
4	80	0.0011
5	90	0.0012
6	100	0.0013

**Table 3:** The calculation results of rolling element life of angular contact ball bearing with radial force of 150 N and axial force of 40 N.

Radial Clearance (μm)	Contact Angle (Degree, Radian)	Equivalent Load (kN)	Fatigue Life (h)
12.4	13.9 (0.2426)	0.0655	14,254
14.4	15 (0.262)	0.0655	19,698
15.3	15.5 (0.2705)	0.0655	21,660
16.5	16.1 (0.2810)	0.0655	24,254
17.2	16.4 (0.2862)	0.0655	25,611
17.8	16.7 (0.2915)	0.0655	27,045
18.4	17.0 (0.2967)	0.0655	28,502
18.7	17.1 (0.2985)	0.0655	29,016
18.9	17.2 (0.3002)	0.0655	29,513
19.3	17.4 (0.3037)	0.0655	30,552
19.5	17.5 (0.3054)	0.0655	31,049
20.0	17.7 (0.3089)	0.0655	32,119
20.2	17.8 (0.3107)	0.0655	32,675
20.4	17.9 (0.3124)	0.0655	33,213
20.7	18.0 (0.3142)	0.0655	33,781
20.9	18.1 (0.3159)	0.0655	34,323

**Table 4:** Results of mechanical properties of angular contact ball bearings with different axial forces.

Axial Load (N)	Maximum Contact Load (kN)	Maximum Contact Stress (Inner Race) /MPa	Maximum Contact Stress (Outer Race) /MPa	Maximum Contact Area Length (Inner Race) /mm	Maximum Contact Area Length (Outer Race) /mm	Contact Angle (Degree)	Fatigue Life (Hour)
40	0.07	1557	1331	0.9	0.8	13.9	21,500
50	0.07	1501	1293	0.9	0.7	15.5	21,500
55	0.06	1472	1258	0.9	0.7	16.1	21,500
60	0.06	1461	1249	0.9	0.7	16.4	21,200
65	0.06	1456	1245	0.9	0.7	16.7	18,700
70	0.06	1455	1244	0.9	0.7	16.7	16,600
75	0.06	1461	1249	0.9	0.7	17.0	14,800
80	0.06	1469	1256	0.9	0.7	17.1	13,200
90	0.06	1489	1273	0.9	0.7	17.2	10,800
100	0.07	1509	1290	0.9	0.7	17.4	8950
110	0.07	1530	1308	0.9	0.8	17.5	7450
120	0.07	1550	1325	0.9	0.8	17.7	6340
130	0.08	1570	1342	1.0	0.8	17.8	5560
140	0.08	1589	1359	1.0	0.8	17.9	4820
145	0.08	1599	1367	1.0	0.8	18.0	4510
150	0.08	1608	1375	1.0	0.8	18.0	4220
160	0.08	1627	1391	1.0	0.8	18.1	3720
170	0.09	1645	1406	1.0	0.8	18.1	3700

## Data Availability

Not applicable.
